# Development and Validation of an Algorithm to Identify Endometrial Adenocarcinoma in US Administrative Claims Data

**DOI:** 10.1155/2019/1938952

**Published:** 2019-11-03

**Authors:** D. B. Esposito, G. Banerjee, R. Yin, L. Russo, S. Goldstein, B. Patsner, S. Lanes

**Affiliations:** ^1^HealthCore, Inc., Wilmington, DE, USA; ^2^Boston University, Boston, MA, USA; ^3^Pfizer Inc., New York, NY, USA; ^4^New York University School of Medicine, New York, NY, USA; ^5^Inova Health System, Falls Church, VA, USA

## Abstract

**Background:**

Endometrial adenocarcinoma is the most prevalent type of endometrial cancer. Diagnostic codes to identify endometrial adenocarcinoma in administrative databases, however, have not been validated.

**Objective:**

To develop and validate an algorithm for identifying the occurrence of endometrial adenocarcinoma in a health insurance claims database.

**Methods:**

To identify potential cases among women in the HealthCore Integrated Research Database (HIRD), published literature and medical consultation were used to develop an algorithm. The algorithm criteria were at least one inpatient diagnosis or at least two outpatient diagnoses of uterine cancer (International Classification of Diseases, Ninth Revision, Clinical Modification (ICD-9-CM) 182.xx) between 1 January 2010 and 31 August 2014. Among women fulfilling these criteria, we obtained medical records and two clinical experts reviewed and adjudicated case status to determine a diagnosis. We then estimated the positive predictive value (PPV) of the algorithm.

**Results:**

The PPV estimate was 90.8% (95% CI 86.9–93.6), based on 330 potential cases of endometrial adenocarcinoma. Women who fulfilled the algorithm but who, after review of medical records, were found not to have endometrial adenocarcinoma, had diagnoses such as uterine sarcoma, rhabdomyosarcoma of the uterus, endometrial stromal sarcoma, ovarian cancer, fallopian tube cancer, endometrial hyperplasia, leiomyosarcoma, or colon cancer.

**Conclusions:**

An algorithm comprising one inpatient or two outpatient ICD-9-CM diagnosis codes for endometrial adenocarcinoma had a high PPV. The results indicate that claims databases can be used to reliably identify cases of endometrial adenocarcinoma in studies seeking a high PPV.

## 1. Introduction

Incidence rates of endometrial cancer exceed those of other uterine cancers in the United States (US), and have risen steadily over the last decade [1]. Adenocarcinoma of the endometrium is the most common histologic site and type of uterine cancer, and was responsible for an estimated 10,470 deaths in the US during 2016 [2]. Administrative databases are commonly used to study rare conditions such as endometrial cancer. The accuracy of diagnostic codes to identify endometrial adenocarcinoma in administrative databases, however, had not been assessed. One concern is that the available ICD-9-CM diagnostic codes (182.xx) do not differentiate between endometrial cancer and uterine sarcoma. The purpose of this study was to develop and validate an algorithm for identification of endometrial adenocarcinoma using a health insurance claims database.

## 2. Methods

The study was performed using administrative claims from the HealthCore Integrated Research Database (HIRD). The HIRD includes individuals who reside across the entire continental US, and is demographically representative of the commercially insured population. It contains longitudinal medical and pharmacy claims data from health plan members. Member enrollment, medical care (professional and facility claims), outpatient prescription drug use, outpatient laboratory test result data, and healthcare utilization may be tracked over time for health plan members. Since 2006, the database contains more than 70 million individuals with enrollment records describing periods of comprehensive medical and pharmacy benefits.

To identify potential endometrial adenocarcinoma patients, we required at least one inpatient diagnosis or at least two outpatient diagnoses of uterine cancer (International Classification of Diseases, Ninth Revision, Clinical Modification (ICD-9-CM) 182.xx) between 1 January 2010 and 31 August 2014. The two outpatient diagnoses could not occur on the same day, but could be separated by any duration of time, as long as they occurred within the study period. The index date was defined as the date of diagnosis for uterine cancer. We required that women had at least 12 months of continuous health plan eligibility prior to their index date and that they had no history of cancer diagnoses (ICD-9-CM 140.xx—209.xx) during this baseline period.

We identified potential cases for whom medical records were sought to validate the algorithm. For a sample of these patients, we requested a single medical record from a specific provider and redacted personally identifying information. To increase the likelihood that the selected medical record included data required for adjudication, we ranked potential medical record sources as follows: (1) hospitalization with uterine cancer listed as the principal diagnosis; or (2) physician office(s) with more than one claim for uterine cancer (ranked by the decreasing number of visits with an associated ICD-9-CM code for uterine cancer).

Each record was reviewed independently by two clinicians (Dr. Marcela Del Carmen, a gynecologic oncologist, and Dr. Bruce Patsner, a specialist in obstetrics and gynecology) to determine diagnoses and whether events identified by the algorithm represented occurrences of endometrial adenocarcinoma. Reviewers used a structured questionnaire to record key clinical findings (e.g., biopsy results, diagnostic procedures and treatments, stage, and preexisting cancers) and ultimately judged case status (confirmed endometrial cancer, not endometrial cancer, and non-evaluable). For endometrial cancer, we considered the ICD-9-CM code182.xx confirmed when the medical record included documentation of an endometrial cancer diagnosis recorded by a treating healthcare provider, and if there were positive results from an endometrial biopsy, pathology reports, surgical procedure(s), or treatment with medications that the adjudication committee believed were consistent with endometrial cancer. Confirmed cases were patients who met the screening criteria and were confirmed by medical record review to be an incident case of endometrial adenocarcinoma. Unconfirmed cases were patients who met the screening criteria but who did not fulfill the criteria for validation.

We calculated the positive predictive value (PPV) as the proportion of cases identified by the algorithm who were confirmed as true cases. Patients whose records were reviewed but found insufficient to determine case status (e.g., a limited examination for mammography only) were excluded. A 95% confidence interval (CI) for PPV was also calculated using the equation for binomial proportions. We also calculated PPV stratified by age at diagnosis (30–44, 45–64, and 65+ years) and care setting where the algorithm criteria were met (inpatient vs. outpatient) for uterine cancer diagnosis.

This study was approved, and a Waiver of Patient Authorization for medical record review was granted by the New England Institutional Review Board.

## 3. Results

**Figure 1:**
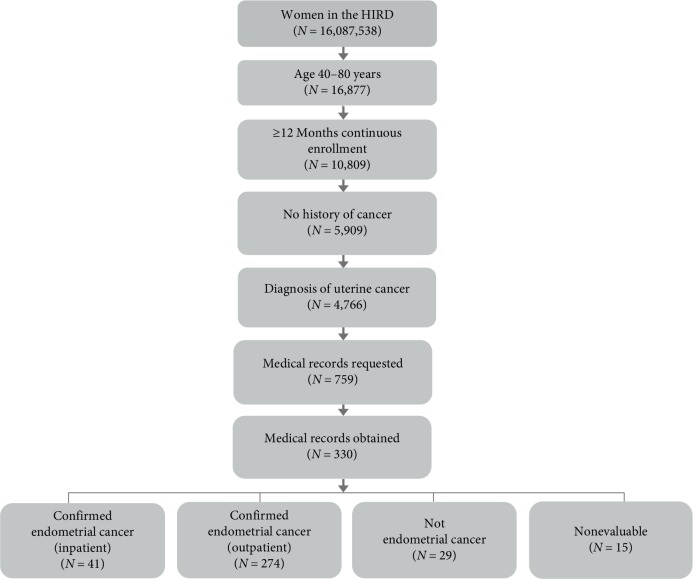
Flowchart of study cohort creation.

We identified 4,766 individuals who met the screening algorithm for endometrial adenocarcinoma between 01 January 2010 and 31 August 2014, and had at least 12 months of continuous baseline enrollment before their index date, and no diagnoses of cancer during this baseline period. Among these women, we selected a random sample of 759 cases as candidates for adjudication by clinical experts (Figure 1). Of these 759 cases, we were able to obtain medical records for 330 (43%) women meeting the algorithm (mean age 63.4 years, standard deviation 10.3). For US census region of residence, 44% resided in the Midwest, 20% in the Northeast, 25% in the South, and 11% in the West. For 53% of the women, medical records were from hospitals, while 47% of the medical records came from ambulatory care (Table 1). We assume that these 330 cases represented a random sample of the 759 cases because the distributions of age and U.S. region of residence were similar to the sample of patients for whom we were unable to obtain medical records.

After clinical adjudication, 286 women were confirmed as having endometrial adenocarcinoma, 29 were classified as having a condition other than endometrial adenocarcinoma, and 15 were found non-evaluable, resulting in a PPV of 90.8% (95% CI 86.9–93.6). The crude level of overall agreement, unadjusted for chance, between the clinical reviewers was about 93%, with a Cohen's kappa of 0.70. Records were identified as false positives based on endometrial biopsy results that did not indicate endometrial cancer or due to a negative biopsy report. Records were considered unevaluable due to missing documentation from biopsy or pathology reports. Of the 29 false-positive cases, the majority were identified as endometrial hyperplasia (17%), 7% of them were endometrial stromal sarcoma or ovarian cancer, and 3% were identified as rhabdosarcoma of the uterus, fallopian tube cancer, leiomyosarcoma, colon cancer, hip problems, a chest mass, or a hysterectomy. Finally, 34% of false positive cases did not have an identifiable alternative diagnosis. Among women who were identified as having uterine cancer using the two outpatient diagnoses criteria, the PPV was 91.6% (95% CI 88.3–94.8). The average duration between two outpatient diagnoses was 50.8 days, with a standard deviation of 119.9 days and a median of 10.5 days. The PPV for women who were identified as having uterine cancer using a single inpatient diagnosis was lower, 85.4% (95% CI 74.5–96.2) (Table 2). The PPV among women who were 65 years of age and older was higher [91.8% (95% CI 87.8–95.8)], than the PPV observed for women who were between 30 and 44 years old, and women who were between 45 and 65 years old (Table 2).

## 4. Discussion

To our knowledge, this is the first study to validate an algorithm to identify endometrial adenocarcinoma in a US administrative claims database. We calculated PPVs as a measure of accuracy of observed uterine cancer claims codes in the HIRD. However, other measures of validity, including sensitivity and specificity, could not be calculated from our data because our study sample included only patients with codes for the diagnosis of interest. Overall, the HIRD contains a large, relatively healthy, working population. Performance of the endometrial cancer algorithm may vary in other populations, particularly with a different prevalence of endometrial cancer.

In many situations, researchers will seek an algorithm with a high PPV [3]. The administrative claims algorithm to identify endometrial adenocarcinoma had an overall PPV of 91%, indicating that about 9% of cases identified in claims were not confirmed by medical record review. We found that our algorithm performed better among women we identified using outpatient claims and among older women, aged 65 years and older. Despite ICD-9-CM diagnosis codes that do not differentiate between endometrial adenocarcinoma and other related diagnoses, owing to the relative rarity of other uterine cancers, such as uterine sarcoma, use of these codes in administrative claims appears useful for identifying endometrial adenocarcinoma. We did not include ICD-9-CM 179.xx, for malignant neoplasm of uterus, parts unspecified, in the algorithm based on the advice of clinicians participating in the adjudication process. As such, the algorithm may have not detected any cases receiving this nonspecific code, possibly sacrificing some sensitivity. Such a trade-off (increased specificity relative to sensitivity) may be appropriate for studies seeking high PPV (e.g., comparative studies with a ratio measure of effect) [3, 4]. Case identification is still imperfect, however, and can vary by treatment group in comparative studies. Therefore, studies that use this or similar algorithms in administrative claims should consider quantitative bias analysis methods to assess the impact of misclassification on results [5].

Sensitivity could also be incomplete in other ways. Any cases with only one outpatient diagnosis or no diagnoses of uterine cancer would not be identified by the algorithm. (We found that of the 5,909 women who we screened for a potential endometrial cancer diagnosis using the algorithm criteria of at least one inpatient diagnosis or at least two outpatient diagnoses of uterine cancer, approximately 4.3% of women had only one outpatient diagnosis of uterine cancer during the whole study period). This could occur due to individuals with endometrial adenocarcinoma not being diagnosed, or terminating employment and, therefore, health coverage due to worsening health status after a first outpatient diagnosis (thus failing to meet the criteria of at least two outpatient diagnoses or one inpatient diagnosis).

When considering application of this algorithm to other study populations, it should be recognized that PPV can vary by study setting (e.g., prevalence) [6]. As such, the PPV observed in this study may not be identical when applying the algorithm in other settings or study populations where endometrial adenocarcinoma is more or less common. In addition, diagnosis patterns and prevalence of types of uterine cancers could change over time. Because the HIRD contains few low-income individuals in the United States, caution is warranted in assuming that algorithm performance would translate to a population covered by US Medicaid.

Finally, future validation will be needed to assess performance of new diagnosis codes and coding system such as the International Classification of Diseases, Tenth Revision (ICD-10). Forward mapping of ICD-9-CM 182.xx results to ICD-10 approximate conversions that are more granular may offer higher specificity (ICD-10 C54.1—malignant neoplasm of endometrium, C54.2—malignant neoplasm of myometrium, C54.3—malignant neoplasm of fundus uteri, and C54.9—malignant neoplasm of corpus uteri unspecified). Backward mapping of the most specific code (C54.1) captures only ICD-9-CM 182, so it is unlikely that we would be picking up more false positives based on the coding transition alone.

## 5. Conclusions

This study offers an estimate of accuracy for claims-based identification of endometrial adenocarcinoma in studies that include data that have been coded in the ICD-9-CM system.

## Figures and Tables

**Table 1:** Characteristics of the study cohort *N* = 330.

Characteristic	Patients (%)
*Age (years)*	
Mean (standard deviation), median	63.4 (10.3), 62
Category	
<40	1.4
40–49	8.1
50–59	26.1
60–69	32.1
70–79	21.4
≥80	10.9

*US region of residence*	
Midwest	44.0
Northeast	20.3
South	24.8
West	10.9

*Type of medical record reviewed*	
Hospitalization	53.4
Ambulatory care	46.6

**Table 2:** Positive predictive values for endometrial cancer *N* = 330.

	Confirmed endometrial cancer	Not endometrial cancer	Unevaluable	PPV	95% CI
Overall	286	29	15	90.8%	86.9–93.6

*Location of claims diagnosis*					
Outpatient	251	23	12	91.6%	88.3–94.9
Inpatient	35	6	3	85.4%	74.5–96.2

*Age group*					
30–44 years	8	1	0	88.9%	68.4–99.9
45–64 years	110	13	8	89.4%	84.0–94.9
65+ years	168	15	7	91.8%	87.8–95.8

## Data Availability

The HealthCore Integrated Database data used to support the findings of this study have not been made available because the authors do not have permission to share data.

## Additional Points

*Prior Postings or Presentations.* Study results were presented at the 2016 International Conference of Pharmacoepidemiology (ICPE) in Dublin, Ireland.
